# Scurvy presenting with limp and weakness: a case report

**DOI:** 10.1186/s12887-019-1605-5

**Published:** 2019-07-06

**Authors:** Robin M. Lund, Mara L. Becker, Steven Shapiro, Tyler Allison, Julia G. Harris

**Affiliations:** 1 University of Louisville School of Medicine, 571 S. Floyd Street, Suite 412, Louisville, KY 40202 USA; 2 Duke Clinical Research Institute, 200 Morris Street, Durham, North Carolina 27701 USA; 3 Children’s Mercy Kansas City, 2401 Gillham Road, Kansas City, MO 64108 USA

**Keywords:** Scurvy, Difficulty walking, Muscle weakness

## Abstract

**Background:**

Scurvy is one of the oldest diseases known to mankind. Although presently rare in the developed world, scurvy was a common potentially fatal disease. In recent times, the most common risk factors for scurvy include alcoholism, low socioeconomic status, and severely poor nutrition or dietary restriction secondary to psychiatric illness or developmental disorders. Our case demonstrates the importance of having a high index of clinical suspicion of an uncommon disease in developed countries and emphasizes the necessity of a dietary screening that could potentially reduce extensive work-up in patients with nonspecific complaints.

**Case presentation:**

We report a case of a 3-year-old previously healthy female originally seen in the rheumatology clinic for limp. She developed weakness and was admitted to the hospital for further evaluation. She underwent extensive diagnostic testing including blood work, magnetic resonance imaging, lumbar puncture, electromyogram, and nerve conduction studies. Ultimately, her vitamin C level returned undetectable. She had immediate and complete improvement upon starting vitamin C supplementation.

**Conclusions:**

Despite being developmentally appropriate, our patient’s refusal to eat fruits or vegetables had limited her diet, emphasizing the importance of obtaining a diet history in a child presenting with an unknown diagnosis. In addition, our patient had no other characteristic features of scurvy, which further supports the need to consider this diagnosis in a child presenting with lower extremity weakness or abnormal gait.

## Background

Scurvy is characterized by a deficiency of vitamin C, also known as ascorbic acid, a water-soluble micronutrient responsible for the production of collagen and carnitine, and the biosynthesis of catecholamines [1]. Humans lack the gene for L-gulonolactone oxidase, an enzyme that converts glucose into ascorbic acid. Thus, vitamin C is an essential exogenous vitamin that when deficient results in myriad symptoms first described in 1550 BC in the Papyrus of Ebers [2–4]. From the world’s first controlled clinical trial in 1753 to determine the role of citrus in the treatment of scurvy, to the isolation and identification of ascorbic acid in 1931, vitamin C deficiency is now easily preventable and treatable [4].

There are many cases of scurvy in the literature primarily involving children with severe dietary restrictions secondary to developmental delay or autism spectrum disorder [2, 5–8]. The clinical manifestations of scurvy are vast. Scurvy can be diagnosed with laboratory testing or with empiric ascorbic acid supplementation, which yields rapid clinical recovery. Despite the ease of treatment, scurvy frequently goes undetected for extended periods of time and can even be fatal. It requires an astute physician to recognize the signs and symptoms and consider the diagnosis of scurvy, especially in developed countries where many physicians have never encountered this disease.

## Case presentation

A 3-year-old previously healthy girl was seen in the rheumatology clinic for a persistent limp of 3 weeks’ duration. There was no known history of trauma. She was able to bear weight but reported right knee pain exacerbated by walking and climbing stairs. She had no pain at rest and no nighttime awakenings. She had no fevers, rashes, rhinorrhea, cough, sore throat, vomiting, or diarrhea. The patient was a “picky eater,” but she was growing appropriately and had met all of her developmental milestones. After the limp had persisted for 2 weeks, she had seen her pediatrician, who performed a right hip radiograph, complete blood count with differential, comprehensive metabolic panel, and erythrocyte sedimentation rate. These were unremarkable except for microcytic anemia with a hemoglobin level of 10.2 g/dL (mean corpuscular volume [MCV] 73 fL, red cell distribution width [RDW] 16%).

At the rheumatology visit, her weight was at the 46th percentile, and her height was at the 83rd percentile. Her vital signs were within normal limits. She had a normal examination of her eyes, mouth, throat, neck, lungs, heart, abdomen, and skin. Her musculoskeletal exam demonstrated normal strength and no tenderness to palpation of her lower extremities. She had intermittent guarding with movement of her right knee but no erythema, warmth, tenderness, limited range of motion or swelling to this joint; the remainder of her joint exam was normal. She had an antalgic gait with minimal right knee movement and favored the right side. Neurologic examination revealed normal mental status, cranial nerve exam, tone, and patellar deep tendon reflexes.

The differential at that time was broad and included infectious, mechanical, inflammatory, oncologic, neurologic, and intra-abdominal etiologies. Initial laboratory work-up included a complete blood count with differential, peripheral smear, and C-reactive protein, which was remarkable for a hemoglobin level of 10.2 g/dL (MCV 70.2 fL, RDW 14.8%) (Table 1). Peripheral smear showed mild microcytic hypochromic anemia without blasts or abnormal cells. A radiograph of her right lower extremity demonstrated mild osteopenia, and a right hip and knee ultrasound were normal. She was discharged home on a non-steroidal anti-inflammatory medication with a plan to obtain additional imaging as an outpatient.

**Table 1:** Select laboratory results

Laboratory test	Value
White blood cell count	5.46 × 10^3^/μL
Hemoglobin	10.2 g/dL
Platelet count	253 × 10^3^/μL
Creatinine	0.36 mg/dL
Aspartate aminotransferase	42 u/L
Alanine aminotransferase	31 u/L
Lactate dehydrogenase	746 u/L
Creatine kinase	230 u/L
Aldolase	4.5 u/L
Erythrocyte sedimentation rate	11 mm/hr
C reactive protein	0.6 mg/dL
Thyroid stimulating hormone	4.1 mIU/L
Vitamin D 25-OH	32 ng/mL
Iron	22 μg/dL
Total iron-binding capacity	481 μg/dL
Transferrin saturation	5%
Ferritin	5 ng/mL
Vitamin C	< 0.1 mg/dL

Over the next 7 days, the patient developed progressive weakness. Parental videos showed the patient exhibiting a Gowers’ sign and having difficulty ascending stairs. Given this acute worsening, she was admitted to the hospital. On admission her strength was abnormal; she would brace herself against furniture to stand and had a positive Gowers’ sign. Direct strength testing against resistance was difficult to obtain, but knee extension, plantar flexion, dorsiflexion and toe flexion were 4/5 bilaterally. She had a wide-based gait and had difficulty fully lifting her feet off the ground while ambulating. Otherwise, her neurological exam was normal regarding her mental status, cranial nerves, deep tendon reflexes, sensation, coordination, and upper extremity strength. Complete metabolic panel, creatine kinase, aldolase, lactate dehydrogenase, uric acid, thyroid stimulating hormone, vitamin D level, and acetylcholine receptor binding antibody were all within normal limits (Table 1). A brain magnetic resonance imaging (MRI) was normal, and a spine MRI was remarkable for a small amount of fat within the filum terminale. Lumbar puncture showed normal cerebrospinal fluid protein, mildly low glucose (48 mg/dL), 1 white blood cell, no red blood cells, and negative enterovirus. MRI of the lower extremities demonstrated increased signal and enhancement of the right paraspinal musculature and bone marrow edema within the right sacral ala, posterior acetabulae, and distal femoral metaphyses. Electromyogram and nerve conduction studies were normal. Plasma amino acids, urine organic acids, acylcarnitine profile, ammonia, and homocysteine were normal. Iron studies demonstrated low iron (22 μg/dL), elevated total iron-binding capacity (481 μg/dL), and low transferrin saturation (5%) (Table 1).

On hospital day 6, her vitamin C level, which had been obtained upon admission in light of her having a restricted diet, returned as undetectable at < 0.1 mg/dL. Upon further questioning, the patient refused to eat fruits or vegetables; her diet at home consisted of dairy products, bread, and crackers. She was started on ascorbic acid 100 mg 3 times daily and had improved strength within 24 h of her first dose. She was discharged shortly thereafter with a diagnosis of scurvy. She returned to her baseline within 2 to 3 weeks. At her follow-up neurology and rheumatology visits, she had a normal neurologic exam including strength and gait. Laboratory results 11 weeks post-discharge demonstrated improving hemoglobin level (11.3 g/dL; MCV 73.7 fL, RDW 13.8%) and normal vitamin C level (1.1 mg/dL). She saw a dietician, who recommended dietary and behavioral modifications and continuation of ascorbic acid supplementation.

## Discussion

Scurvy is a preventable disease caused by poor intake and/or absorption of vitamin C. Fruits and vegetables are the primary dietary source of this essential vitamin. Typically, the first symptoms of scurvy are nonspecific, including fatigue, anorexia and weight loss; these appear after 60 to 90 days of a diet lacking ascorbic acid [4, 9, 10]. Cutaneous findings, including petechiae, ecchymoses, hyperkeratosis, corkscrew hairs, and perifollicular hemorrhage, can develop after about 5 months of a diet deficient in vitamin C and are frequently found in the lower extremities because the capillaries also face hydrostatic pressure [4, 9, 10]. Many cutaneous manifestations are secondary to blood vessel fragility as ascorbic acid is paramount in collagen synthesis [4]. In the gingiva, this is seen as erythema, swelling, and teeth loss [4]. Anemia is common and partially due to iron deficiency, because vitamin C aids in iron absorption. Additional manifestations can include lower extremity edema, splinter hemorrhages, alopecia, poor wound healing, cardiac hypertrophy, psychological changes, and conjunctival hemorrhages, among many others [4].

Pediatric patients commonly present with musculoskeletal manifestations. With damage to synovial blood vessels and microfractures, there is pain and swelling secondary to subperiosteal hemorrhages and hemarthroses [9]. Osteoporosis may develop within the trabeculae and cortical bone, sites of endochondral ossification, which require collagen to develop a strong osteoid matrix [11]. Additionally, vitamin C deficiency increases bone resorption [9].

Case reports of musculoskeletal manifestations with scurvy are often accompanied by classic skin or mucosal changes. A case series from Thailand reported that 96% of children diagnosed with scurvy presented with inability to walk [12]. In this cohort, 96% also had limb pain, 43% had gingival bleeding, 46% had lower extremity joint swelling, 36% had gum hypertrophy, and 3.6% had petechial hemorrhage. Another report highlights a 12-year-old boy with scurvy who presented with leg pain and refusal to bear weight [2]. However, in contrast to our child, he had global developmental delay, skin findings, and mucosal changes. Similarly, another case report highlights a 9-year-old patient with progressive leg weakness and musculoskeletal pain, but examination was also pertinent for gum hypertrophy, mucosal bleeding, ecchymosis, and hyperkeratosis [13]. There are additional reports of infants presenting with pseudoparalysis due to presumed leg pain [11, 12] and patients presenting with joint swelling [14, 15]. The lack of other symptoms in our patient may be due to her disease being in an early stage or due to a mild form of the disease.

Pediatric scurvy frequently has classic radiographic findings, which were not seen in our patient. These findings include cortical thinning, zone of provisional calcification at the metaphysis (lines of Fränkel), lucent metaphyseal band (Trümmerfeld zone), metaphyseal spurs (Pelkan spurs), or calcification around the epiphysis (Wimberger ring sign) [1, 5, 11, 14]. In a case series of 3 children diagnosed with scurvy who presented with difficulty walking, all patients had radiographs containing lines of Fränkel [1]. In another case report involving a child who had difficulty walking, radiographs demonstrated lines of Fränkel and metaphyseal spurs [14]. Our patient’s radiographs showed only osteopenia, the most common yet nonspecific finding in scurvy [5, 11]. Our patient also had an abnormal MRI demonstrating areas of increased bone marrow signal, which is nonspecific but was reported in all four cases in the largest case series of MRI findings in pediatric scurvy [6]. Furthermore, this case series noted signal abnormalities centered in the metaphyses, and the distal femoral metaphyses were involved in our patient.

Given the dietary deficiency needed to cause scurvy, this disease is mainly seen in elderly people, alcoholics, and food faddists [4, 9]. Scurvy is less common in the pediatric population, with most reports in patients with severe dietary restrictions secondary to developmental delay, autism, or psychiatric disorders [2, 5–8]. Other pediatric risk factors include ketogenic diet, chronic total parenteral nutrition usage, malignancy, poor intestinal absorption, food allergies, or infants fed boiled milk [9–11, 16].

## Conclusions

Our case is notable given our patient was developmentally normal without autistic characteristics. In addition, she presented solely with an abnormal gait and weakness without other characteristic findings of scurvy, resulting in an extensive neurologic work-up. Our case highlights the importance of obtaining a dietary history in patients with unknown diagnoses. We recommend administration of empiric ascorbic acid while awaiting lab verification if the history is concerning for poor vitamin C intake. In addition, our case emphasizes the need to consider scurvy as a possible, albeit rare, diagnosis for a child with unexplained limp or lower extremity weakness.

## Data Availability

Not applicable.

## Abbreviations

MCV: Mean corpuscular volume
MRI: Magnetic resonance imaging
RDW: Red cell distribution width
